# ERG K^+^ channels mediate a major component of action potential repolarization in lymphatic muscle

**DOI:** 10.1038/s41598-023-41995-5

**Published:** 2023-09-09

**Authors:** Hae Jin Kim, Min Li, Emma C. Erlich, Gwendalyn J. Randolph, Michael J. Davis

**Affiliations:** 1 Department of Medical Pharmacology & Physiology, University of Missouri, One Hospital Drive, MA415 Medical Sciences Building, Columbia, MO 65212 USA; 2 Department of Pathology and Immunology, Washington University, St Louis, MO USA

**Keywords:** Physiology, Cardiovascular biology

## Abstract

Smooth muscle cells in the walls of collecting lymphatic vessels fire spontaneous action potentials (APs), which conduct rapidly over the muscle layer to initiate contractions that propel lymph. Several ion channels have been implicated in the currents underlying the AP spike and the preceding diastolic depolarization, but the molecular identities of K^+^ channels involved in AP repolarization are unknown. Based on previous studies of other rhythmically active smooth muscles, we hypothesized that ether-a-go-go related gene (ERG) K^+^ channels (Kv11) play an important role in repolarization of the AP in lymphatic muscle. Message for one or more ERG channel isoforms was detected by RT-PCR analysis of lymphatic vessels from mice, rats and humans. Membrane potential recordings in smooth muscle cells of rat and human lymphatics revealed that nanomolar concentrations of ERG-1 inhibitors (E-4031 and BeKm-1) prolonged the duration of the AP plateau (normally ~ 1 s in duration) and induced multiple spikes, whereas ERG-1 activators (ICA-105574 and RPR-260243) shortened the plateau and could completely inhibit spontaneous APs. At relatively high inhibitor concentrations, the AP plateau duration lasted as long as 24 s. ERG activators reversed the effects of ERG inhibitors and vice-versa. In pressure myograph studies, ERG channel inhibition prolonged the diastolic repolarization phase of the contraction cycle and reduced the frequency of spontaneous contractions. This is the first evidence for a specific K^+^ channel contributing to the AP in lymphatic muscle. Our results imply that lymphatic contractile dysfunction may occur in long QT type II patients with mutations that result in ERG channel loss-of-function or impaired trafficking of the channel to the cell membrane.

## Introduction

Collecting lymphatic vessels generate spontaneous, twitch-like contractions that, in concert with a one-way valve system to prevent backflow, enable lymph to be actively transported against the adverse hydrostatic pressure gradients that develop in dependent extremities^1,2^. Indeed, active lymphatic pumping accounts for two thirds of lymphatic transport in the lower legs of humans during quiet standing^3,4^. Lymph transport is matched to the filling state of the lymphatic vessel network in large measure because the contraction frequency of the lymphatic muscle cell (LMC) layer in collecting vessels is exquisitely sensitive to changes in luminal pressure^5^.

Lymphatic muscle exhibits both rhythmic contractions and agonist-modulated basal tone, thereby sharing characteristics of both cardiac muscle and vascular smooth muscle. LMCs fire spontaneous action potentials (APs), which are rapidly conducted over the LMC layer to initiate contractions^6–8^. Lymph propulsion is optimized by the relatively high ejection fraction of lymphatic collectors (often approaching 0.8) and contraction waves that are entrained over lengths of one or more lymphangions. The membrane potential (Vm) of LMCs is not stable but exhibits a gradual, spontaneous depolarization (i.e., pacemaking potential) during the diastolic phase of the lymphatic contraction cycle, bringing Vm to a threshold that triggers firing of an AP^9,10^. Lymphatic pacemaking potentials are generated by the interaction of a number of ion channels, including voltage-gated sodium channels^9,11,12^, the calcium-activated Cl^-^ channel anoctamin-1^13^, hyperpolarization-activated, cyclic nucleotide-gated (HCN) channels^14–16^, and possibly other non-selective cation channels^10,17^. The relative contributions of these channels vary between species, but in all cases depolarization to threshold triggers the activation of L-type voltage-gated channels (VGCCs), which carry most or all inward current for the upstroke of the lymphatic AP, and mediate the majority of Ca^2+^ entry responsible for LMC contraction^18–21^.

A single AP typically initiates a single LMC twitch contraction, after which repolarization initiates another cycle of slow depolarization. Voltage-gated K^+^ currents presumably set the resting Vm and facilitate AP repolarization, and previous studies have shown that Vm and outward K^+^ currents in LMCs are sensitive to millimolar concentrations of the K^+^ channel inhibitors tetraethylammonium (TEA) and 4-aminopyridine (4-AP)^9,22,23^. However, these compounds are broad inhibitors of many K^+^ channels^24^ and the molecular identities of the K^+^ channels involved in LMC repolarization have not been determined.

Twelve families of voltage-gated K^+^ (Kv) channels have been identified molecularly^25^, most of which are expressed in various types of smooth muscle^26^. Of these, the properties of Kv11 (for ether-a-go-go-related channel, and hereafter referred to as ERG) channels, which exhibit rapid voltage-dependent inactivation and recovery from inactivation, are particularly suited to controlling the duration of the AP plateau, as demonstrated in a substantial literature on cardiomyocyte ion channels^27^. Because ERG channels, and the ERG1 isoform (Kv11.1) in particular, are expressed in certain types of smooth muscle that normally generate spontaneous APs and exhibit rhythmic contractile activity, we hypothesized that they may play an important role in LMCs. Indeed, ERG channels contribute to AP repolarization in smooth muscle cells of the myometrium^28–30^, bladder^31^, esophagus^32^, portal vein^33^ and gall bladder^34^, and the latter has an AP shape (in rat) that is nearly identical to the APs recorded in rat mesenteric LMCs^10,35^. While delineation of the roles of various Kv channel isoforms in a given cell type can be difficult, ERG1 channels can be distinguished by their electrophysiological signature under voltage-clamp conditions and by their sensitivity to relatively selective ERG1 channel inhibitors and activators (with IC_50_ values in the nanomolar range). The goal of the present study was to use these pharmacologic tools to determine whether functional ERG channels are expressed in LMCs and contribute to the resting Vm and/or AP repolarization.

## Results

### ERG message is expressed in lymphatic muscle

**Figure 1:**
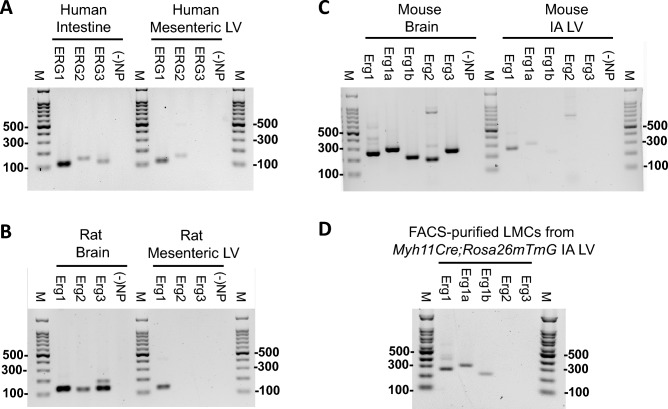
RT-PCR analysis of ERG channel message in human, rat and mouse lymphatic vessels. (**A**) Human *ERG1* = 131 bp, *ERG2* = 150 bp, *ERG3* = 123, 145 bp detected in samples of intestinal wall (for reference) or isolated mesenteric lymphatic vessels. (**B**) Rat *Erg1* = 131 bp, *Erg2* = 124 bp, *Erg3* = 129,153 bp detected in brain (for reference) or isolated mesenteric lymphatic vessels. (**C**) Mouse *Erg1* = 263 bp, *Erg1a* = 303 bp, *Erg1b* = 219 bp, *Erg2* = 194 bp, *Erg3* = 267 bp detected in brain (for reference) or isolated inguinal-axillary lymphatic vessels. (**D**) Mouse *Erg1* = 263 bp, *Erg1a* = 303 bp, *Erg1b* = 219 bp, *Erg2* = 194 bp, *Erg3* = 267 bp detected in FACS-purified (GFP +) LMCs from isolated inguinal-axillary lymphatic vessels of *Myh11CreER*^*T2*^*; Rosa26mTmG* mice. Each gel is representative of 3 separate samples. All images were obtained using Image Lab™ Software Version 3.0 from BIO-RAD.

RT-PCR was used to test for ERG channel message in segments of rat, mouse and human lymphatic vessels. There are three genes, *KCNH2*, *KCNH6* and *KCNH7,* that correspond to the genes *ERG1, 2 and 3*. For the purposes of this manuscript we use the classical gene terms *ERG1-3*. Human mesenteric lymphatics consistently expressed message for ERG1 and ERG2, as shown in Fig. 1A. Rat mesenteric lymphatics and mouse inguinal-axillary lymphatics expressed message only for ERG1 (Fig. 1B,C). To further examine if ERG message was derived from LMCs rather than, or in addition to, another cell type in the lymphatic vessel wall, we purified LMCs from lymphatic vessels dissected from *Myh11CreER*^*T2*^*;Rosa26mTmG*^*f/f*^ mice. After thorough cleaning of individual inguinal-axillary collectors, the vessels were pooled and digested. The single cell suspension was sorted by FACS and GFP+ cells were collected and tested for ERG isoform expression using RT-PCR^36^. This analysis confirmed that *ERG1* was expressed in LMCs (Fig. 1D) and we identified message for both *ERG1a* and *1b* variants in mouse. These analyses reveal that ERG1, which controls AP plateau duration in cardiomyocytes and other cell types, is expressed in lymphatic vessels from all three species.

### ERG inhibitors delay LMC repolarization and prolong lymphatic diastole

**Figure 2:**
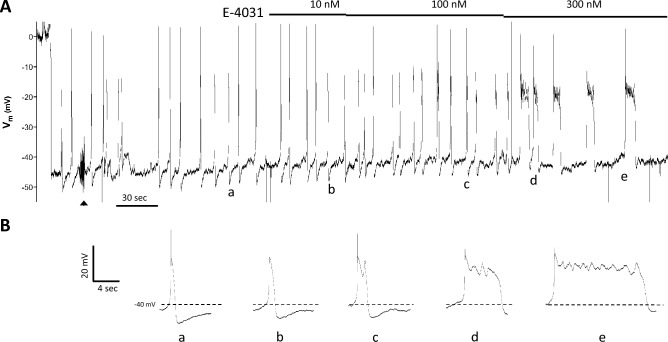
Vm recordings from the LMC layer of a rat pressurized mesenteric lymphatic vessel in response to the ERG1 inhibitor E-4031. (**A**) After the initial impalement, Vm dropped rapidly from 0 to − 46 mV and spontaneous APs fired with spikes (in most cases) to + 2 mV. Adjustment of the electrode position and resistance test (at arrowhead) during the first 90 s of recording caused a transient increase in AP frequency that subsequently stabilized. After that time, E-4031 was applied in cumulative doses at the indicated times and concentrations. (**B**) Expanded time scale shows the shape of individual APs (**a–e**) at the corresponding times marked (**a–e**) in (**A**).

Next, we investigated whether APs in LMCs were sensitive to the widely used ERG1 channel inhibitor, E-4031^27,31,33^. Of the three species—rat, mouse and human—mouse LMCs were the most difficult from which to make successful Vm measurements. Difficulties with obtaining successful recordings from human lymphatics limited experimentation in that species to a few Vm recordings (see Suppl. Methods). Thus, the majority Vm measurements were recorded from LMCs of pressurized rat mesenteric lymphatic collectors. A representative Vm recording from the LMC layer of a rat mesenteric lymphatic vessel is shown in Fig. 2. After impalement, there was a rapid drop in Vm to ~  − 46 mV, followed by a short period of stabilization. Insert (a) shows the shape of a typical AP in a rat LMC, with a resting Vm ~ -42 mV, gradual depolarization in diastole to a threshold for AP initiation of ~  − 40 mV, a single spike to ~ 0 mV, followed by a plateau at ~ -15 mV lasting 1.0 s, ending with rapid repolarization and after-hyperpolarization to ~ -50 mV. Subsequent application (b) of the ERG1 inhibitor E-4031 (10 nM final bath concentration) led to a slight widening of the AP plateau (to 1.4 s). 100 nM E-4031 caused a slight depolarization (~ 2 mV) and further widening of the plateau (to 3.4 s) along with a blunted second spike (c). 300 nM E-4031 greatly extended the plateau duration (to 7.0 s), which contained multiple blunted spikes (d). These effects were even more exaggerated in some APs (e). Higher concentrations of E-4031 sometimes caused a sustained depolarization that abolished APs altogether (not shown).

**Figure 3:**
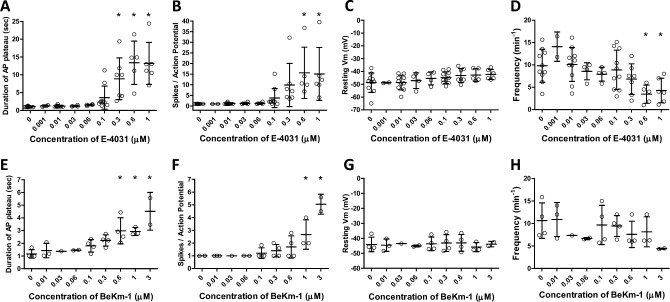
Summary data showing the effects of the ERG1 inhibitors E-4031 and BeKm-1 on various components of the AP in LMCs of rat mesenteric lymphatics. (**A**) The duration of the AP (defined as the time from the peak of the spike to the point at which Vm repolarized to its initial threshold level) increased with increasing concentration of E-4031; IC_50_ = 0.24 μM. (**B**) The number of “spikes” per AP (defined as fluctuations > 3 mV during the plateau phase) also increased with increasing concentration of E-4031; IC_50_ = 0.25 μM. (**C**) There was a trend for resting Vm to depolarize with increasing concentrations of E-4031, reaching ~ 6 mV at 1 μM, but this effect was not statistically significant. (**D**) AP frequency decreased as the concentration of E-4031 increased, with significance reached at the two highest concentrations. (**E–H**) Summary effects of BeKM-1 on the duration of the AP plateau (**E**; IC_50_ = 0.92 μM), the number of spikes per AP (**F**; IC_50_ = 1.3 μM), resting Vm (**G**) and frequency (**H**). *Significantly different from control value using a one-way ANOVA with Dunnett’s post-hoc tests, p < 0.05. Error bars are ± SD. N = 5, n = 10.

Summary data for the effects of E-4031 on APs of rat mesenteric LMCs are shown in Fig. 3. Increasing concentrations of E-4031 caused progressive widening of the AP plateau, measured as the length of time from the spike to the point during repolarization when Vm crossed the original resting Vm; the IC_50_ was 240 nM and concentrations above 100 nM produced plateau durations that were statistically increased from control (Fig. 3A). Likewise, E-4031 led to an increase in the number of spikes per AP (Fig. 3B), with IC_50_ = 250 nM; concentrations above 300 nM were statistically different from control. When high concentrations of E-4031 were applied to naïve LMCs, larger secondary spikes were often observed (Suppl. Fig. 1). No significant changes in AP plateau duration were observed during a separate series of experiments testing for time and/or vehicle effects (Suppl. Fig. 2). Increasing concentrations of E-4031 produced progressive depolarization (Fig. 3C), reaching ~ 6 mV at 1 μM E-4031, but these changes were not statistically significant. Increasing concentrations of E-4031 caused reductions in the frequency of AP firing (Fig. 3D). The magnitude of the after-hyperpolarization was also reduced by E-4031 in some cells (see Fig. 2 for an example), but this effect was not statistically significant.

We also tested the effects of the peptide ERG channel inhibitor, BeKm-1, which works through a different mechanism of action than E-4031. While E-4031 works an open channel blocker, possibly binding to the S6 domain^37^, BeKm-1 is an ERG-specific toxin that binds near the pore to stabilize the close state^38^. BeKm-1 also increased the duration of the AP plateau (Fig. 3E) and increased the number of spikes per AP (Fig. 3F), albeit at higher concentrations than E-4031 (IC_50_ = 0.92 and 1.3 μM, respectively). BeKm-1 did not produce significant effects on resting Vm (Fig. 3G) or the magnitude of the after-hyperpolarization (not shown). Increasing concentrations of BeKm-1 caused reductions in the frequency of AP firing (Fig. 3H), although the differences were not statistically significant.

**Figure 4:**
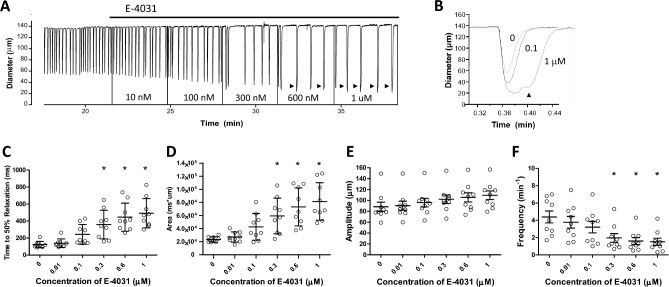
Effects of E-4031 on contractions of rat pressurized mesenteric lymphatics. (**A**) E-4031 increased the amplitude and decreased the frequency of spontaneous contractions. Vertical lines (to Diam = 0) reflect transient blanking of the light path to mark addition and mixing of each concentration E-4031. (**B**) E-4031 prolonged the diastolic relaxation phase of the contraction cycle and higher concentrations were often associated with double contractions (also marked arrowheads in **A**), which were extremely rare under normal conditions in rat mesenteric lymphatics. (**C–F**) Summary of E-4031 effects on time to 50% relaxation (**C**), area under the diameter vs. time curve (**D**), contraction amplitude (**E**) and frequency (**F**). *Significantly different from control value using ANOVA with Dunnett’s post-hoc tests, p < 0.05. Error bars are ± SD. N = 4, n = 9.

In parallel protocols using pressurized rat mesenteric lymphatic vessels in which diameter, rather than Vm, was measured (in the absence of wortmannin), E-4031 significantly altered several components of diastole. An example is shown in Fig. 4A. At this compressed time scale, the most obvious effect of E-4031 was to decrease the contraction frequency by increasing the time between contractions, but analyses of single, twitch contractions at higher time resolution (Fig. 4B) revealed substantial increases in the durations of the twitch contractions, primarily due to increases in the time required for relaxation. Systole was also prolonged, contributing to the increase in contraction duration and leading to an increase in contraction amplitude. In some cases, there were two components to the twitch contraction (arrowheads in Fig. 4A and expanded traces in Fig. 4B; see also the diameter trace in Suppl. Fig. 1A), events which were extremely rare under normal conditions. Summary data in Fig. 4C–F showed that ERG1 inhibition significantly increased the time to half maximal relaxation (Fig. 4C) and the area under the diameter-time curve (Fig. 4D); EC_50_ = 150 and 200 nM, respectively, as estimated from curve fits in IGOR. E-4031 slightly increased the contraction amplitude (Fig. 4E) and significantly decreased contraction frequency (Fig. 4F). The reduction in frequency occurred both as a result of an increased time to half maximal relaxation (the early phase of diastole) and an increase in the time between twitch contractions (i.e., the later phase of diastole).

### ERG activators shorten the duration of the AP plateau phase

**Figure 5:**
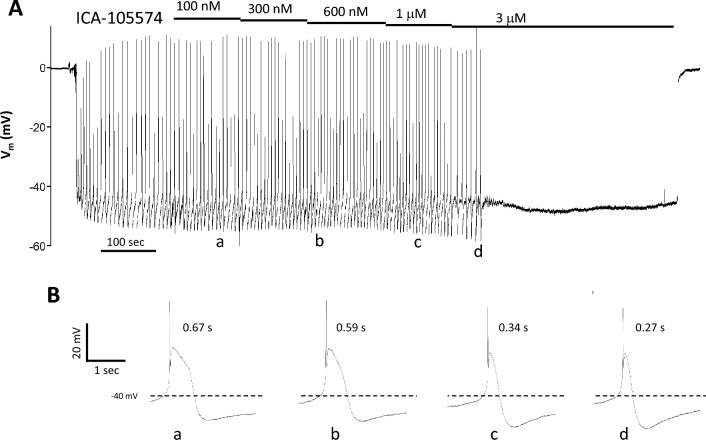
Representative Vm recording from the LMC layer of a rat pressurized mesenteric lymphatic showing the effects of ICA-105574. (**A**) The entire recording, showing the initial impalement, the cumulative effects of five concentrations of ICA-105574, the slowing of frequency at 1 μM ICA-105574, the eventual cessation of spontaneous APs at 3 μM ICA-105574 and the return to 0 mV when the electrode is pulled out of the cell. (**B**) Expanded time scales show how the duration of the plateau phase of the AP is shortened with increasing concentrations of ICA-105574. Individual APs marked (**a–d**) correspond to the times marked (**a–d**) in panel (**A**).

**Figure 6:**
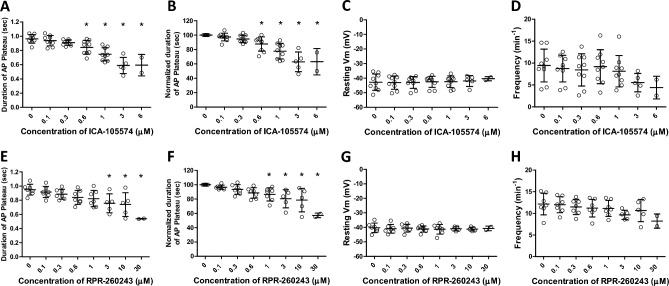
Summary of effects of the ERG channel activators ICA-105574 and RPR-260243 on Vm of rat pressurized mesenteric lymphatics. (**A,B**) ICA-105574 decreases the duration of the AP plateau phase, and the effect is equally significant if the duration is normalized to the initial control value (**B**). ICA-105574 does not significantly alter the resting Vm (**C**), or frequency (**D**), although there is a trend for frequency to decrease. RPR-260243, another ERG channel activator that works through a different mechanism of action, produces a similar effect on the plateau duration (**E,F**) but requires higher concentrations to reach significance. RPR does not significantly affect the resting Vm (**G**) or frequency (**H**), although there is a trend for frequency to decrease. *Significantly different from control value using a one-way ANOVA with Dunnett’s post-hoc tests, p < 0.05. Error bars are ± SD. N = 5, n = 9.

Next, we tested the effects of the ERG channel activator, ICA-105574^27^, which attenuates ERG inactivation by shifting its voltage dependence to more positive potentials^39^. Increasing concentrations of ICA-105574 resulted in progressive narrowing of the AP plateau. An example recording is shown in Fig. 5 in which the ultimate effect of 3 μM ICA-105574 was to decrease the width of the AP plateau to ~ 30% of control prior to complete cessation of spontaneous APs. In some cells, ICA-105574 also appeared to increase the magnitude of the after-hyperpolarization (see example in Fig. 5Bd), but analysis of the summary data indicated that this effect was not statistically significant. The effects of ICA-105574 and another ERG channel activator RPR-260243, which works by slowing the kinetics of ERG channel deactivation^40^, are summarized in Fig. 6. Both activators produced a decrease in the duration of the AP plateau (Fig. 6A,E; EC_50_ =  ~ 800 nM and 2 μM, respectively). No significant changes in AP plateau duration were observed during a separate series of experiments testing for time and/or vehicle effects (Suppl. Fig. 3). Neither activator produced a statistically significant increase in the resting Vm (Fig. 6C,G), or frequency (Fig. 6D,H), although in both cases there was a trend for frequency to decrease with increasing activator concentration.

### ERG activators and inhibitors have counteracting effects on the lymphatic AP

**Figure 7:**
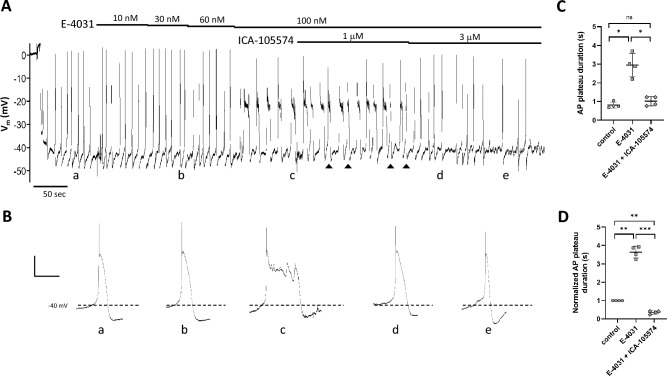
ICA-105574 reverses the effects of E-4031. (**A**) After impalement and Vm stabilization, bath application of E-4031 leads to progressive widening of the AP plateau such that at time point (**c**) there are multiple secondary “spikes”. The subsequent addition of 1 μM ICA-105574 in the continued presence of E-4031 leads to the narrowing of some (arrowheads) but not all APs, whereas 3 mM ICA-105574 consistently reduces plateau duration (**e**) to values lower than control (**a**). (**B**) Expanded time scales show how the duration of the plateau phase of the AP is altered first by the addition of E-4031 and then subsequent addition of ICA-105574. Individual APs marked (**a–e**) correspond to the times marked (**a–e**) in (**A**). Calibration bar: 10 mV·2 s^−1^ for all traces except (**c**) where it is 10 mV·5.8 s^−1^. (**C,D**) Summary data for 4 vessels from 2 animals, using either the raw (**C**) or normalized (**D**, to control) data for AP plateau duration. The concentration of E-4031 was 100 nM; the concentration of ICA-105574 was 3 μM. Error bars are ± SD. Significance determined using a one-way ANOVA with Tukey’s multiple comparison tests. *Indicates p < 0.05; **indicates p < 0.01; ***indicates p < 0.001; ns = not significant at p < 0.05.

**Figure 8:**
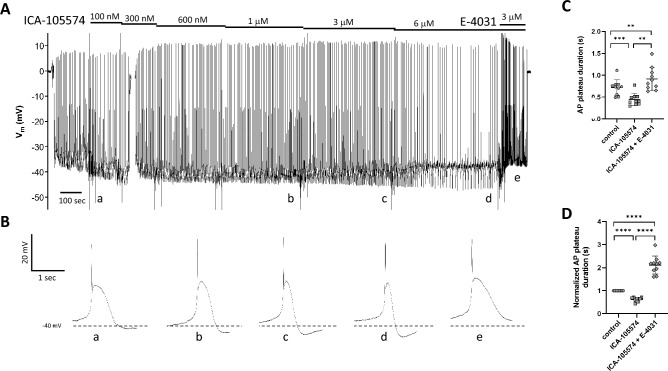
E-4031 reverses the effects of ICA-105574. (**A**) After impalement and Vm stabilization, bath application of increasing concentrations of ICA-105574 leads to progressive narrowing of the AP plateau with eventual slowing of frequency. The subsequent application of E-4031 (3 μM) increases the frequency and results in widening of the AP plateau to a value slightly wider than control. (**B**) Expanded time scales show how the plateau phase of the AP changes first in response to ICA-105574 and then to ICA-105574 + 3 μM E-4031. Individual APs marked (**a–e**) correspond to the times marked (**a–e**) in (**A**). Note: for the “control” AP, an early recording in 100 nM ICA-105574 was selected because Vm had not completely stabilized before the first application of ICA-105574. Recording is representative of 3 similar experiments. Summary data for 10 vessels from 7 animals, using either the raw (**C**) or normalized (**D**, to control) data for AP plateau duration. Error bars are ± SD. Significance determined using a one-way ANOVA with Tukey’s multiple comparison tests. **Indicates p < 0.01; ***indicates p < 0.001; ****indicates p < 0.0001.

We also tested whether ERG channel activators and inhibitors would antagonize each other in their effects on the lymphatic muscle AP. Representative recordings of this protocol are shown in Figs. 7 and 8. As before, the application of E-4031 led to an increase in the duration of the AP plateau (Fig. 7A), which became particularly evident at 100 nM E-4031 (Fig. 7Bc). Subsequent addition of ICA-105574 (3 μM, but not 1 μM) in the continued presence of E-4031, reversed the effects of the inhibitor, shortening the duration of the plateau to the extent that it was even less than control (Fig. 7Be; 0.73 s for control, 0.45 s in 100 nM E-4031 + 3 μM ICA-105574). Summary data for AP plateau duration and normalized AP plateau duration are given in Fig. 7C,D. An example of the reverse protocol is shown in Fig. 8. Here, ICA-105574 was applied in increasing concentrations, again leading to progressive narrowing of the AP plateau at 1, 3 and 6 μM (Fig. 8Bc-d), with plateau width at 6 μM being reduced to 44% of control. Subsequent application of E-4031, in the continued presence of ICA-105574, led to an increase in the frequency of spontaneous APs and a plateau duration that was 27% greater than control. Summary data for AP plateau duration and normalized AP plateau duration are given in Fig. 8C,D.

### ERG inhibitors prolong AP duration and diastole in human lymphatic muscle

Lastly, we obtained two recordings of Vm in wire myograph-mounted human lymphatic vessels in response to multiple concentrations of E-4031 that showed the same pattern of response as in rat mesenteric LMCs, i.e., prolongation of the AP plateau with the appearance of multiple spikes. One of those recordings is shown in Suppl. Fig. 4, where the AP plateau duration increased from 1.7 s (control) to 5.7 s, with multiple spikes, after application of 1.2 μM E-4031. In addition, the effects of E-4031 on the diameter of a pressurized human mesenteric vessel were similar to those observed in rat mesenteric vessels, as confirmed in the example shown in Suppl. Fig. 5. Increasing concentrations of E-4031 prolonged the duration of the contraction phase and were associated with an increase in the number of “double” contractions (arrowheads, Suppl. Fig. 5A), in which second contractions were often initiated before the vessel had relaxed to the previous end diastolic diameter (Suppl. Fig. 5B). Increasing concentrations of E-4031 led to progressive increases in the half time to relaxation (Suppl. Fig. 5C), increases in area under the diameter-time curve (Suppl. Fig. 5D), increases in contraction amplitude (Suppl. Fig. 5E), and decreases in frequency (Suppl. Fig. 5F). The similarities between the responses to E-4031 of human and rat vessels in both Vm (Fig. 2, Suppl. Fig. 4) and diameter recordings (Fig. 3, Suppl. Fig. 5) suggest that hERG channels in human LMCs play similar roles in AP repolarization as in rodent LMCs.

## Discussion

Our results support the conclusion that ERG1 (Kv11.1) channels mediate a major component of AP repolarization in lymphatic muscle. This is the first report of a specific K^+^ channel contributing to AP repolarization in this cell type. ERG1 channel message is expressed in mouse, rat and human lymphatic vessels, and FACS sorting of murine lymphatic vessels expressing a GFP reporter in the smooth muscle layer show that ERG1 is present in LMCs. Vm measurements in rat and human vessels reveal that ERG1 inhibitors delay AP repolarization and ERG1 activators accelerate repolarization, with IC_50_ and EC_50_ values in the nanomolar range. In pressurized mesenteric lymphatics from rats and humans, the ERG1 inhibitor E-4031 has clear effects on the relaxation phase of spontaneous contractions, lengthening the time required for relaxation and decreasing the frequency of spontaneous contractions. We conclude that ERG channels play a critical role in the repolarization of the AP generated by LMCs. Loss of ERG channel activity results in prolonged AP duration and a lower frequency of active lymphatic pumping, which are predicted to lead to a net impairment in lymph transport.

The role of ERG1 channels in controlling AP repolarization and contraction frequency in LMCs is consistent with their documented contribution to the rhythmicity of other types of smooth muscle. The spontaneous APs of rat gall bladder smooth muscle have nearly identical characteristics to the APs in rat LMCs shown in Figs. 2, 5, 7 and 8: a resting Vm between − 40 and − 45 mV, a single AP spike to + 5 mV, an AP plateau lasting ~ 1 s and spontaneous firing that is initiated by gradual diastolic depolarization^34^. In gall bladder, E-4031 had very similar effects (at somewhat higher concentrations) to what we observed in rat LMCs: prolongation of the AP plateau, induction of multiple spikes, delayed repolarization, increased contraction amplitude and decreased frequency^34^. ERG channels also contribute to the spontaneous oscillations in Vm and/or contractility in bladder^31^, esophagus^32^, jejunum^41,42^, portal vein^33,43^, epididymis^44^ and myometrium^28,29^. In mouse and human myometrium, ERG1 activity decreases in late pregnancy, due to upregulation of inhibitory accessory KCNE channel β-subunit(s)^28,29^, which reduce ERG activity by increasing the rate of channel deactivation^45^. Unlike some other types of smooth muscle^32–34,43^, ERG1 inhibitors had no significant effect on the resting Vm of LMCs, suggesting that ERG1 channels are not strongly activated until an AP spike is initiated. However, we did observe E-4031-mediated depolarization in some LMCs (Fig. 2) and more consistently so if naïve cells were exposed to high, rather than gradually increasing, concentrations of the inhibitor (Suppl. Fig. 1). In gall bladder, a clear depolarizing effect of E-4031 was recorded when APs were first blocked by diltiazem^34^, but we did not test that protocol.

It is interesting that the application of both ERG1 activators and inhibitors led to a decrease in the frequency of LMC APs and lymphatic vessel contractions. Explaining the effect of the inhibitors on frequency is straightforward: lengthening the AP plateau duration from 1 to 24 s (in the case of 300 nM E-4031 in Fig. 2Be) must necessarily result in a lower AP and contraction frequency. Although ERG1 activators accelerated repolarization and narrowed the AP plateau width, higher concentrations of ICA-105574 lowered both AP and contraction frequency by reducing overall LMC excitability, which is evident in the traces shown in Figs. 5 and 7, where diastolic depolarization was slowed or reversed, such that Vm underwent multiple oscillations before reaching threshold. This reduced overall excitability would account for the trend in Fig. 6 for frequency to be progressively reduced as the concentrations of the ERG-1 activators increased.

Although the inhibitors and activators used in our protocols are reported to be selective for Kv11 channels at low concentrations^37,38^, we are unable to completely rule out contributions from other Kv channel family members—Kv1 in particular—to the observed effects of these agents on AP repolarization in LMCs. TEA and 4-AP were found in previous lymphatic studies to increase the duration of the AP plateau in rat LMCs, but these compounds produced substantial depolarization and repetitive AP firing compared to E-4031^22,23,46^. The differences in the pattern of responses to TEA and 4-AP (Suppl. Fig. 6) vs. ~ 10,000-fold lower concentrations of E-4031 (Fig. 4A) suggest that E-4031 does not exert its effects on AP plateau duration in LMCs by inhibiting other Kv isoforms. A way to resolve this issue would be to perform voltage-clamp studies of single, isolated LMCs. ERG1 current is most definitively distinguished in voltage-clamp protocols of K^+^ tail currents by an initial ‘hook’ that results from partial inactivation during depolarization and which is removed (reversed) with a fast time course by hyperpolarization^27,28,33,47^. This rapid voltage-dependent inactivation and recovery from inactivation presumably makes the ERG channel well suited to determine the duration of the plateau phase of an AP^27^. Although such protocols were beyond the scope of the present study, future experiments utilizing voltage-clamp protocols on single, enzymatically isolated LMCs could be used to identify the ERG1 current signature, determine the sensitivity of that current to ERG1 inhibitors/activators, and to determine the fraction of whole-cell K^+^ current that is sensitive to concentrations of ERG1 inhibitors/activators. Additional electrophysiology studies in transgenic mice, e.g., Vm recording and/or patch clamp protocols in mouse LMCs, could test the impact of conditional ERG1 deletion on the AP shape and the frequency and amplitude of spontaneous contractions.

ERG-1 currents can contribute to pacemaking if they are active at the resting Vm and their slow deactivation after an AP promotes diastolic depolarization. This property has been demonstrated in SA nodal cells^48–52^, but it could also facilitate pacemaking potentials in other cell types, including LMCs. Again, the potential role of ERG1 channels in lymphatic pacemaking would best be studied using voltage or current clamp protocols in LMCs and in mice with conditional knock out of ERG1 channels. The contribution of ERG channels to LMC pacemaking is predicted to depend on the resting Vm and thus may vary between lymphatics of different body regions and between species, as Vm appears to be more negative in human than rat or mouse lymphatic vessels^10,13,35,36,53^.

ERG-1 channels mediate the rapid component of the delayed rectifier current controlling AP repolarization in cardiac myocytes and ERG1 mutations account for about one fourth of all hereditary arrhythmias in humans, including class II long QT syndrome. Because ERG1 channels exert similar control over the AP plateau duration in LMCs, is likely that the same ERG1 mutations also affect lymphatic contractile function, as well as the function of other tissues/organs with rhythmically active smooth muscle. Our data show that the loss of ERG channel activity produces a substantial prolongation of the AP plateau duration in LMCs, resulting in a reduction in the normal frequency of spontaneous lymphatic contractions. These effects are predicted to impair both active lymphatic pumping and lymph transport, which is a question that could be explored in clinical studies.

## Methods

### Animal procedures

All procedures were carried out in accordance with relevant guidelines and regulations as approved by the animal care committee at the University of Missouri and complied with the standards stated in the “Guide for the Care and Use of Laboratory Animals” (National Institutes of Health, revised 2011). The study is reported in accord with ARRIVE guidelines.

### Human tissues

Protocols on human lymphatics were conducted as approved by the Human Research Protection Office at Washington University (protocol #201111038 to GJR) and conformed to the principles of the Declaration of Helsinki and with relevant guidelines and regulations. Informed consent was obtained from all subjects and/or their legal guardian(s). Discarded samples of mesenteric fat and sometimes gut wall were collected from de-identified consenting patients undergoing intestinal surgical resections for bowel inflammation, obstruction, cancer or polyps at Washington University Barnes-Jewish Hospital. Staff affiliated with the Washington University Digestive Diseases Research Core Center obtained the informed consent, provided tissue collection oversight, and de-identification services as per protocol.

### Mice and rats

Male Sprague–Dawley rats, 180–200 g, were obtained from Envigo (Indianapolis, IN). Male or female C57Bl/6J mice were obtained from JAX (JAX: 000664) and used at 2–3 months of age. For FACS analyses, *Myh11-CreER*^*T2*^ mice (B6.FVB-Tg(*Myh11*-cre/ERT2)1Soff/J) obtained from Stefan Offermanns (Max-Planck Institute, Bad Neuheim) were crossed to *ROSA26mT/mG* reporter mice (JAX: 007676). The male offspring were induced with i.p. injections of 100 mg tamoxifen (10 mg/ml in safflower oil) on five consecutive days. For genotyping, genomic DNA was extracted from tail clips using the HotSHOT method. Genotypes were determined by PCR with 2 × PCR Super Master Polymerase Mix (Catalog # B46019, Bimake, Houston, TX) according to the provider’s instructions. Mice were provided ad libitum access to food and water and housed under normal light and dark cycles in cages of up to five mice.

### Mouse and rat vessel isolation and cannulation

Mice and rats were anesthetized with ketamine/xylazine (100/10 mg/kg, i.p.) and placed face down on a heated pad. Inguinal-axillary lymphatic vessels in the mouse were exposed through a superficial incision in the side, removed and transferred to a dissection chamber filled with Krebs-albumin solution for further dissection. Rat mesenteric lymphatics were isolated by opening the abdomen and removing the entire small intestine, which was then pinned out for dissection of individual collecting vessels. Regardless of the vessel source, a short vessel segment containing at most 1 valve was then transferred to a 3-ml myograph chamber containing Krebs-albumin solution and cannulated at each end with a glass micropipette (60–80 μm OD), pressurized to 3 cmH_2_O, and further cleaned to remove excess fat and connective tissue. The chamber, with associated micropipettes, pipette holders and micromanipulators, was transferred to the stage of an inverted microscope. The micropipettes were connected to either a moveable reservoir for manual control of pressure or a microfluidic controller (Elveflow Instr., Paris) for computer control of pressure. Custom LabVIEW programs (National Instruments; Austin, TX) acquired analog data from low pressure sensors connected to the controller, simultaneously with vessel inner diameter, as detected from video images acquired using a Basler firewire camera^54^. To minimize axial bowing of the vessel at higher intraluminal pressures, inflow pressure (Pin) and outflow pressure (Pout) were briefly set to 10 cmH_2_O at the beginning of every experiment and the segment was stretched axially to remove slack. After the pressures were returned to 3 cmH_2_O, the vessel was allowed to equilibrate in Krebs buffer at 37°C for at least 20 min until spontaneous contractions developed and the contraction pattern stabilized. Constant exchange of buffer was maintained using a peristaltic pump at a rate of 0.5 mL/min.

### Vm recording

Sharp electrode measurements of Vm were made in the muscle layer of rat and human lymphatic vessel segments using SEC-05x (NPI Electronics) or Axoclamp2A (Axon Instruments) amplifiers^55^. Micropipettes were pulled from 1.0/0.5 mm borosilicate glass to tip resistances of 150–300 MΩ when filled with 1 M KCl. The amplifier outputs were digitized at 1 kHz using an A-D interface (National Instr.) and recorded, along with the outputs of the pressure or force transducers, using LabVIEW programs. Short vessel segments (1–2 mm in length), containing at most 1 valve, were used to ensure isopotentiality of the LSM layer. For Vm recording in rat vessels, 2 μM wortmannin was added to the bath to attenuate (but not completely abolish) the contraction amplitude, increasing the likelihood that a stable impalement and recording could be maintained during multiple additions of agonist/antagonist. Wortmannin was not needed for human vessel recordings, which were performed under isometric conditions. For analysis of AP parameters, the digitized Vm recording was imported into IGOR (Wavemetrics, Oswego, OR), and the number of spikes, AP plateau duration, resting Vm and magnitude of after-hyperpolarization were measured for each AP. These parameters were averaged for a single LMC over several APs (the number depended on the spontaneous AP frequency) to obtain a single value for subsequent statistical testing.

### RNA isolation and end-point PCR

Lymphatic vessels were dissected from rat mesentery, human mesentery or mouse flank, and thoroughly cleaned of fat and connective tissue. Total RNA was extracted using the Arcturus PicoPure RNA isolation kit (ThermoFisher Scientific, Waltham, MA) with on-column DNase I treatment (Qiagen, Valencia, CA) according to manufacturer’s instructions. RNA was eluted with nuclease-free water. Purified RNA was transcribed into cDNA using High-Capacity cDNA Reverse Transcription kit (ThermoFisher Scientific). End-point PCR was performed in a reaction mixture containing first-strand cDNA as the template, 2 mM MgCl_2_, 0.25 μM primer, 0.2 mM deoxynucleotide triphosphates; and GoTaq® Flexi DNA polymerase (Promega, Madison, WI). Approximately 50–60 sorted cells were used per reaction and PCR protocols were performed as follows: initial preheating at 95 °C for 5 min, 35 cycles of denaturation (94 °C, 30 s), annealing (58 °C, 30 s), extension (72 °C, 30 s) and a final elongation step for 5 min at 72 °C. The amplified PCR products were loaded on a 1.5% agarose gel by electrophoresis, stained with SYBR-Safe (ThermoFisher Scientific), and visualized by UV transillumination. All primers were designed to amplify intron-spanning DNA regions. Primers are listed in Table 1.

**Table 1:** Information on primer sequences used in Fig. 1.

Gene		Accession#	Strand	Sequence	Amplicon	Exon
Rat						
Kcnh2	Erg1	NM_053949	s	GAT CGC CTT CTA CCG GAA AG	131	2–3
			as	CTA CCA TGT CCT TCT CCA TCA C		
Kcnh6	Erg2	NM_053937	s	TCG CTG TCC ACT ATT TCA AGG	124	5–6
			as	GCC GTC TTC AGT AAC CCA ATC		
Kcnh7	Erg3	NM_131912	s	ATC GAC ATG CCA GTG AAG AC	129	4–6
		XM_032903397	as	GTT GCG GGA TCT TGT TAA TGG	153	
Human						
KCNH2	Erg1	NM_172056	s	CAG TGA CCG TGA GAT CAT AGC	119	5–7
		NM_000238	as	GTG CCT GCA GCT TGT ACT	131	
KCNH6	Erg2	NM_173092	s	CCA ACA CCA ACT CCG AGA AG	150	6–7
		NM_030779	as	CCT TGA CAC GCA GCA TCT G		
KCNH7	Erg3	NM_173162	s	ATA TTT AGA GAC CGA CAT GCC A	123	4–6
		NM_033272	as	GAG TGA GCT GTG GAA TCT TGT	145	
Mouse						
Kcnh2	Erg1a	NM_013569	s	CCC TCC ATC AAG GAC AAG TAT G	263	7–8
	& 1b		as	GGG TTG GGA ATC TGG TGA AA		
Kcnh2	Erg1a	NM_013569	s	AGA ACT GCG CTG TCA TCT AC	303	2–3
			as	CCA TGT CCT TCT CCA TCA CTA C		
Kcnh2	Erg1b	AF_012869	s	CAG GCA GCT GTC CAT ACT C	219	1–2
			as	GAA TCG CCA TGG AGG ACT TAG		
Kcnh6	Erg2	NM_001037712	s	GCC AAT CAG GTG CTG CCC CT	194	10–11
			as	GAG GTC TGA CGA CAC GCG GG		
Kcnh7	Erg3	NM_133207	s	CCA GAC TCC ATG GTG AAG AAA	267	13–14
			as	CCG TTG TCA TTT GGG ATT CAA G		

Amplicon size is in bp.

### FACS analysis

Inguinal-axillary lymphatic vessels from tamoxifen-treated *Myh11CreER*^*T2*^*;Rosa26mTmG* mice were dissected as described previously^55^. Cleaned vessel segments were transferred to a 1-ml tube of low-Ca^2+^ PSS containing (in mM): 137 NaCl, 5.0 KCl, 0.1CaCl_2_, 1.0 MgCl_2_, 10 HEPES, 10 Glucose, and 1 mg/ml BSA at room temperature for 10 min. The solution was decanted and replaced with a similar solution containing 26 U/ml papain (Sigma, St. Louis, MO) and 1 mg/ml dithioerythritol. The vessels were incubated for 30 min at 37 °C with occasional agitation, then transferred to a new tube containing low-Ca^2+^ PSS containing 25 mg/ml collagenase H (FALGPA U/ml, Sigma), 0.7 mg/ml collagenase F (Sigma), 20 mg/ml trypsin inhibitor (Sigma), 1 mg/ml elastase (Worthington), and incubated for 6 min at 37 °C. The cells dispersed from digested vessels were sedimented by centrifugation (300g, 4 min), resuspended in 0.6 ml PSS containing 1mM Ca solution, and filtered through a nylon filter with 35-µm mesh size to obtain single cell suspension. GFP+ smooth muscle cells were collected using fluorescence‐activated cell sorting (FACS) with a Beckman-Coulter MoFlo XDP instrument using an excitation laser (488 nm) and emission filter (530/40 nm), with 70 µm nozzle at a sheath pressure of 45 psi and sort rate of 100 events per second. Sorting was performed at the Cell and Immunobiology Core facility at the University of Missouri.

### Solutions and chemicals

Krebs buffer contained: 146.9 mM NaCl, 4.7 mM KCl, 2 mM CaCl_2_·2H_2_O, 1.2 mM MgSO_4_, 1.2 mM NaH_2_PO_4_·H_2_O, 3 mM NaHCO_3_, 1.5 mM Na-HEPES, and 5 mM D-glucose (pH 7.4). An identical buffer (“Krebs-BSA”) also contained 0.5% bovine serum albumin. Krebs-BSA buffer was present both luminally and abluminally during cannulation, but the abluminal solution was constantly exchanged with plain Krebs during the experimental protocol. For Ca^2+^-free Krebs, 3 mM EGTA replaced CaCl_2_·2H_2_O. All chemicals were obtained from Sigma-Aldrich (St. Louis, MO) except BSA (US Biochemicals; Cleveland, OH), MgSO_4_ and Na-HEPES (ThermoFisher Scientific). E-4031 was obtained from Tocris (#1808) and dissolved in water to make a 1 mM stock solution. BeKm-1 was obtained from Alomone Labs (#STB-470) and dissolved in water to make a 100 mM stock solution. RPR-260243, type 1 ERG channel agonist, was obtained from MedChemExpress (#HY-16915) and dissolved in DMSO to make a 10 mM stock solution. ICA-105574, a type 2 ERG channel agonist, was obtained from Abious (#AOB2681) and dissolved in DMSO to make a 10 mM stock solution.

### Statistics

N refers to the number of animals and *n* refers to the number of vessels or cells included per group; these values are stated in the figure legends. Statistical analyses were performed using Prism (v.9.5.1; Graphpad, San Diego CA). Specific statistical tests are stated in each figure legend as appropriate. Data are plotted as mean ± SD. Unless otherwise stated, the significance level was *p* < 0.05. IC_50_ and EC_50_ values were determined in Prism or, when fits did not converge, estimated from curve fits of the data set in IGOR.

### Supplementary Information

Supplementary Information 1.

Supplementary Figure 1.

Supplementary Figure 2.

Supplementary Figure 3.

Supplementary Figure 4.

Supplementary Figure 5.

Supplementary Figure 6.

Supplementary Information 8.

## Data Availability

Data are available on request from the authors. The data that support the findings of this study are available from the corresponding author upon reasonable request. Some data from human vessels may not be made available because of privacy or ethical restrictions.

## Abbreviations

ERG: Ether-a-go-go
BeKm-1: A 21 amino acid peptide derived from scorpion venom (RPTDIKCSESYQCFPVCKSRFGKTNGRCVNGFCDCF)
E-4031: *N*-[4-[[1-[2-(6-Methyl-2-pyridinyl)ethyl]-4-piperidinyl]carbonyl]phenyl]methanesulfonamide dihydrochloride
ICA-105574: (±)-*trans*-10,11-Dihydroxy-5,6,6a,7,8,12b-hexahydrobenzo[*a*]phenanthridine hydrochloride
RPR-260243: (3R,4R)-4-[3-(6-methoxyquinolin-4-yl)-3-oxo-propyl]-1-[3-(2,3,5-trifluoro-phenyl)-prop-2-ynyl]-piperidine-3-carboxylic acid
TEA: Tetraethylammonium
4-AP: 4-Aminopyridine
