# Supplementary figures and images for: ERG K+ channels mediate a major component of action potential repolarization in lymphatic muscle
Source: Sci Rep. 2023 Sep 9;13:14890. doi: 10.1038/s41598-023-41995-5 (PMC10492848; doi:10.1038/s41598-023-41995-5)

Original Gels for Figure. 1

Figure. 1A

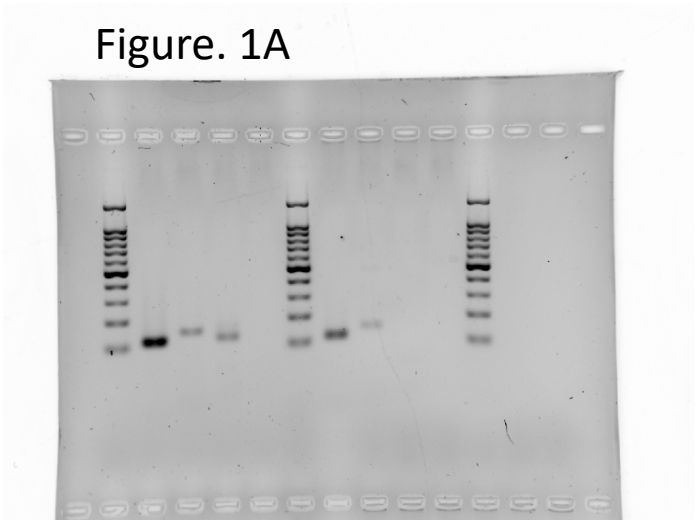

Figure. 1C

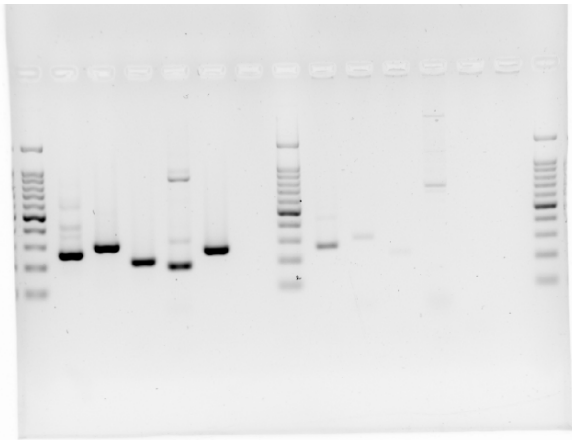

Figure. 1B

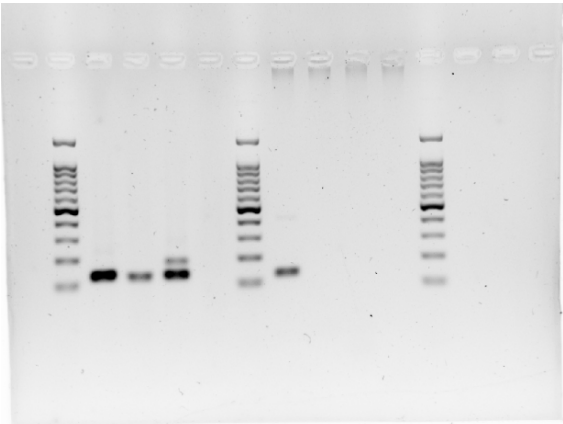

Figure. 1D

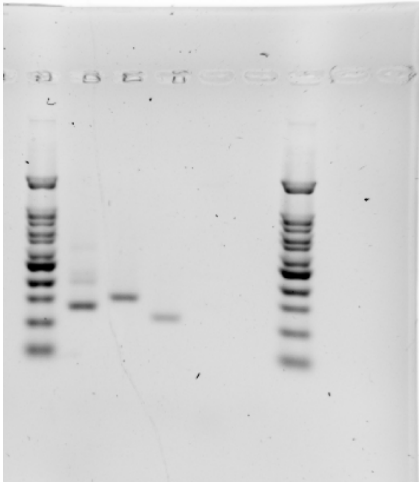

Supplement: Supplementary file 1 — Supplementary Information 1. [file 41598_2023_41995_MOESM1_ESM.pdf]
