# Supplementary material for: ERG K+ channels mediate a major component of action potential repolarization in lymphatic muscle
Source: Sci Rep. 2023 Sep 9;13:14890. doi: 10.1038/s41598-023-41995-5 (PMC10492848; doi:10.1038/s41598-023-41995-5)
Supplement: Supplementary file 2 — Supplementary Figure 1. [file 41598_2023_41995_MOESM2_ESM.pdf]

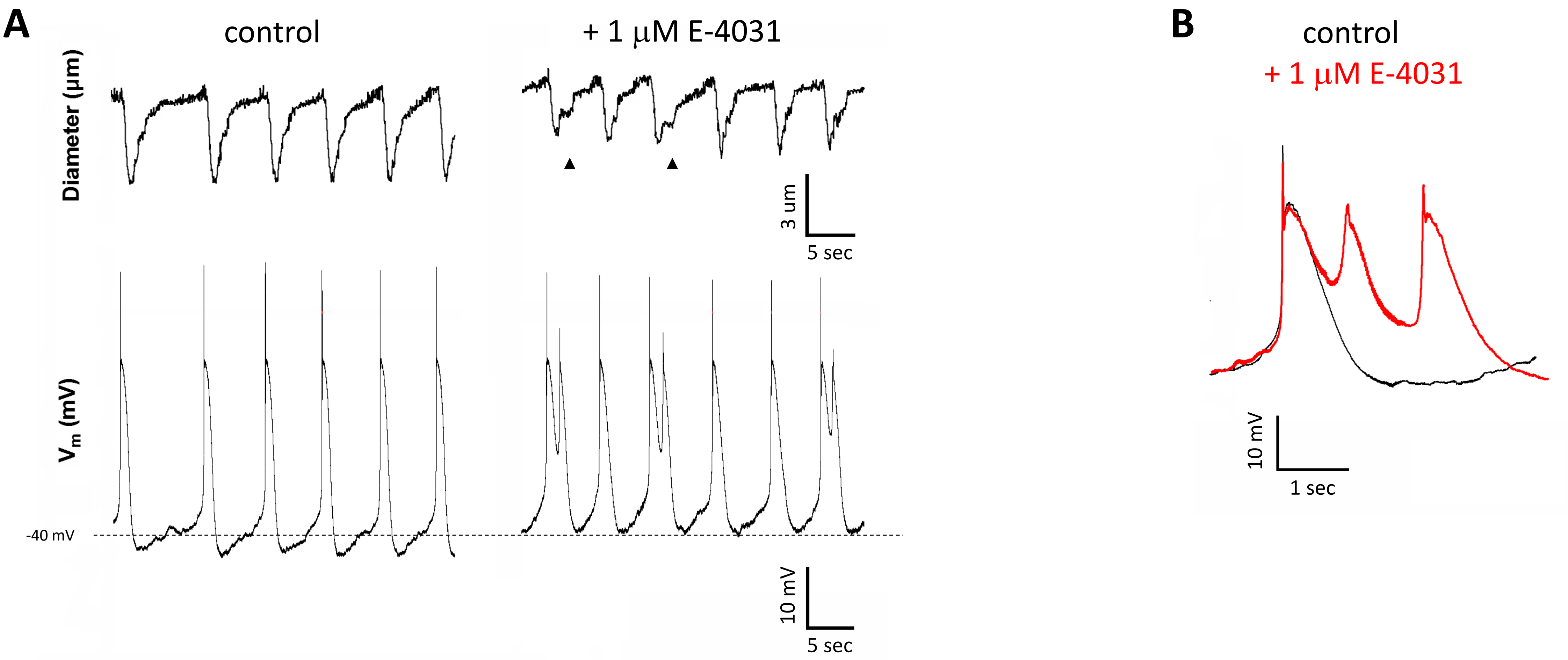

Supplemental Fig. 1. **A**) Vm recording in an LMC along with diameter recording of a rat pressurized mesenteric lymphatic (after wortmannin treatment), showing that 1 μM E-4031 led to double spikes in half of the APs, corresponding in at least two cases with double contractions (arrowheads). **B**) Vm recording in an LMC of another rat pressurized mesenteric lymphatic in which 1 μM E-4031 led to three spikes.
