# Supplementary material for: ERG K+ channels mediate a major component of action potential repolarization in lymphatic muscle
Source: Sci Rep. 2023 Sep 9;13:14890. doi: 10.1038/s41598-023-41995-5 (PMC10492848; doi:10.1038/s41598-023-41995-5)
Supplement: Supplementary file 3 — Supplementary Figure 2. [file 41598_2023_41995_MOESM3_ESM.pdf]

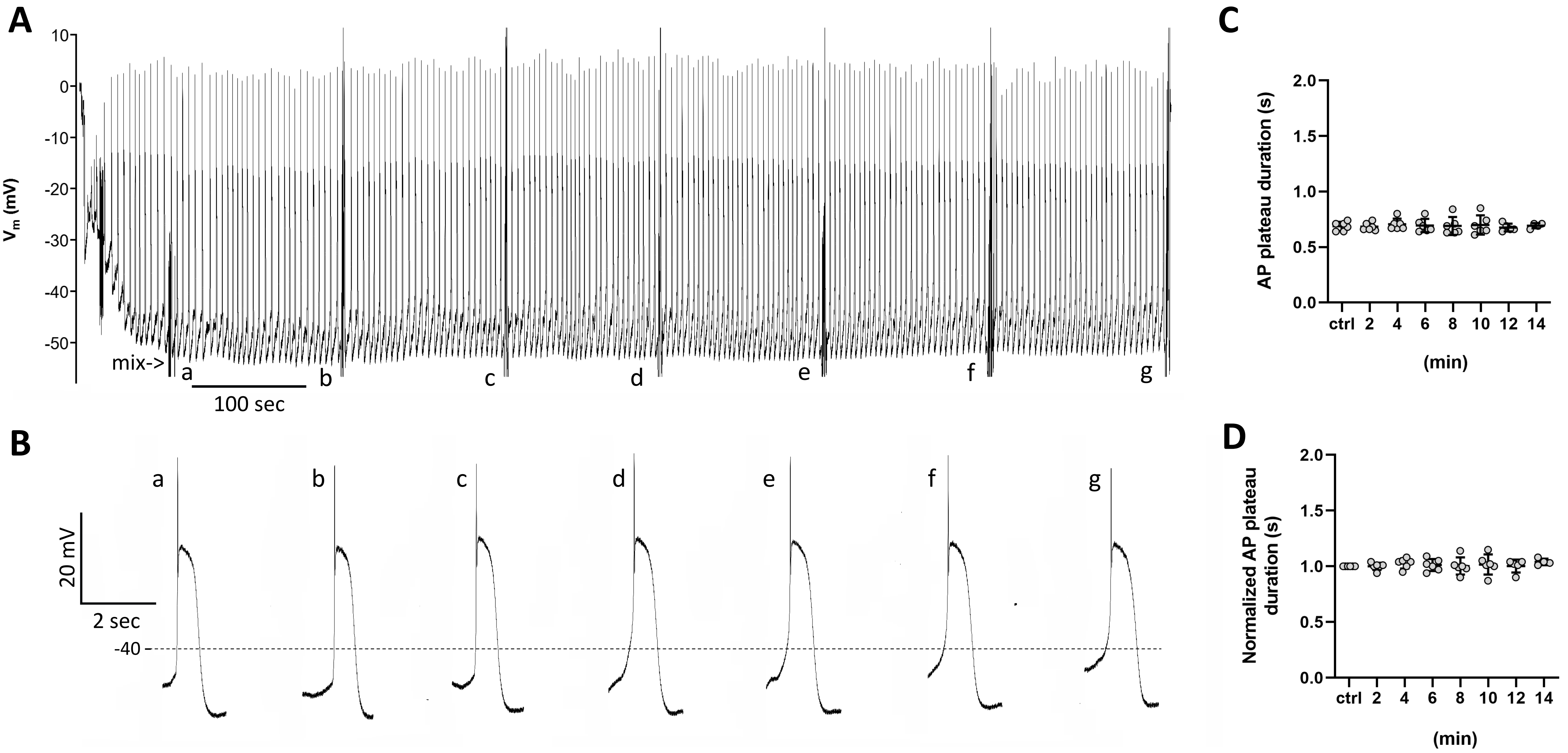

Supplemental Fig. 2. Time and vehicle controls for E-4031 and BeKm-1. **A**) Recording showing time control with addition and mixing of vehicle (Krebs solution without ERG inhibitor) at ~2-min intervals. B) Insets show representative APs at expanded time scale for each time interval. Summary of changes in AP plateau duration (**C**) or normalized AP plateau duration (**D**) over time. Error bars are SD. There were no significant differences from control in either C or D, using a one-way ANOVA with Dunnett's post-hoc tests. N= 4; n = 6.
