# Supplementary material for: ERG K+ channels mediate a major component of action potential repolarization in lymphatic muscle
Source: Sci Rep. 2023 Sep 9;13:14890. doi: 10.1038/s41598-023-41995-5 (PMC10492848; doi:10.1038/s41598-023-41995-5)
Supplement: Supplementary file 4 — Supplementary Figure 3. [file 41598_2023_41995_MOESM4_ESM.pdf]

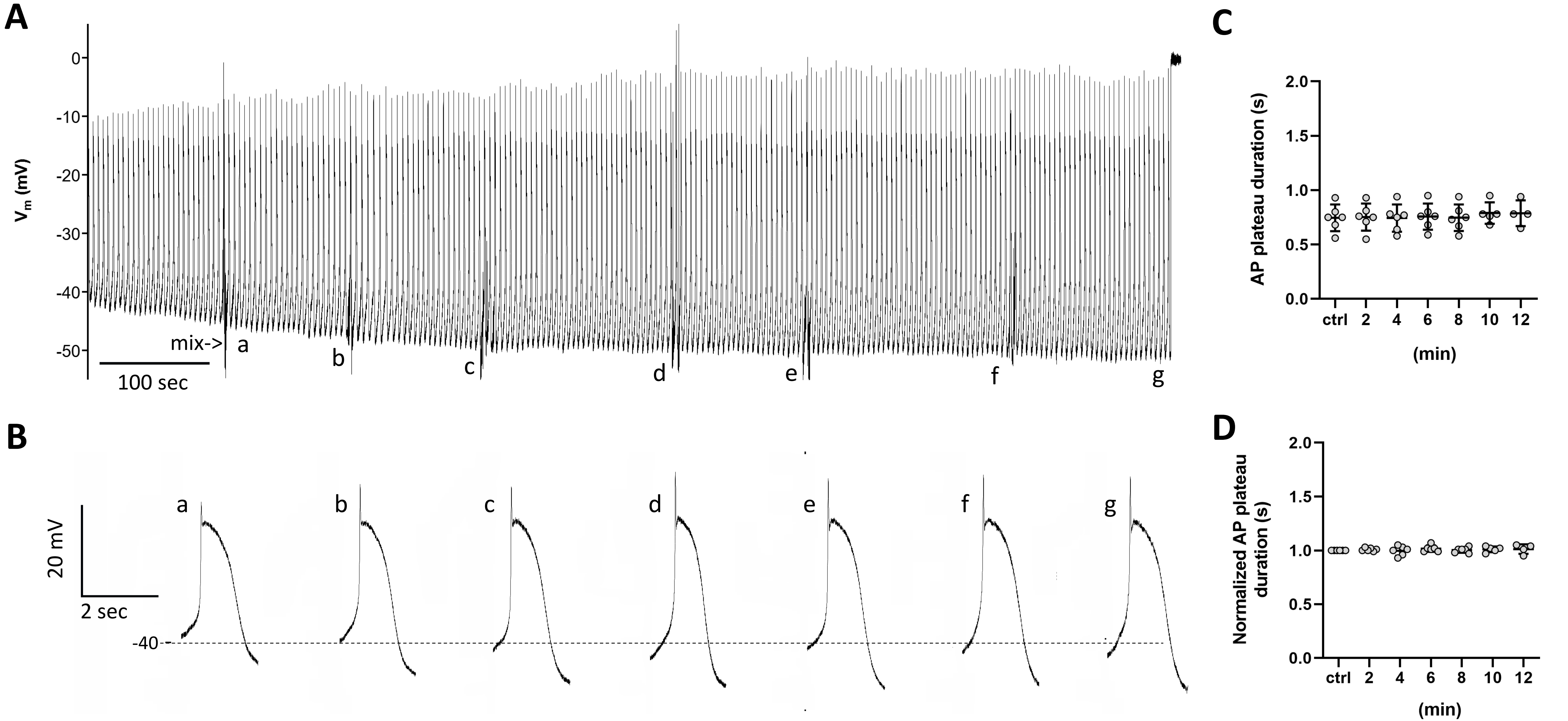

Supplemental Fig. 3. Time and vehicle controls for ICA-105574 and RPR-260243. **A**) Recording showing time control with addition and mixing of vehicle (DMSO, at equivalent concentrations used for the ERG channel activators but without the activator) at ~2-min intervals. **B**) Examples of individual APs on an expanded time scale (a-g) at the corresponding time points indicated in panel A. Summary of changes in AP plateau duration **C**) or normalized AP plateau duration (**D**) over time. Error bars are SD. There were no significant differences from control in either **C** or **D**, using a one-way ANOVA with Dunnett's post-hoc tests. N= 3; n = 6.
