# Supplementary material for: ERG K+ channels mediate a major component of action potential repolarization in lymphatic muscle
Source: Sci Rep. 2023 Sep 9;13:14890. doi: 10.1038/s41598-023-41995-5 (PMC10492848; doi:10.1038/s41598-023-41995-5)
Supplement: Supplementary file 5 — Supplementary Figure 4. [file 41598_2023_41995_MOESM5_ESM.pdf]

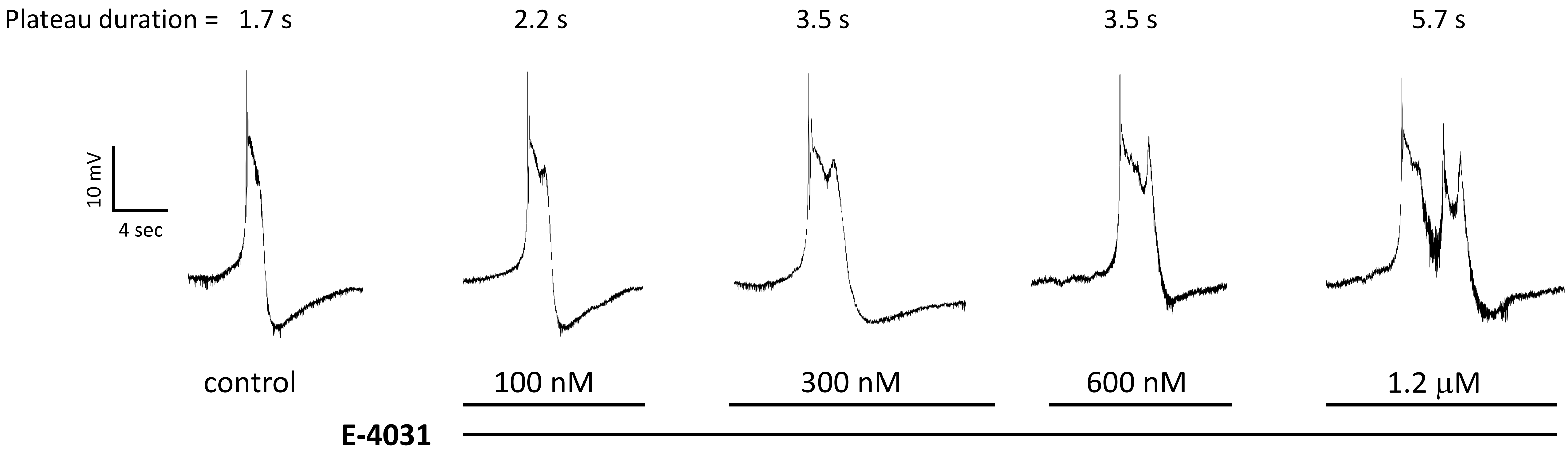

Supplemental Fig. 4. Vm recording from a human pressurized lymphatic vessel showing progressive widening of the AP plateau, with multiple spikes, in response to increasing concentrations of E-4031.
