# Supplementary material for: ERG K+ channels mediate a major component of action potential repolarization in lymphatic muscle
Source: Sci Rep. 2023 Sep 9;13:14890. doi: 10.1038/s41598-023-41995-5 (PMC10492848; doi:10.1038/s41598-023-41995-5)
Supplement: Supplementary file 6 — Supplementary Figure 5. [file 41598_2023_41995_MOESM6_ESM.pdf]

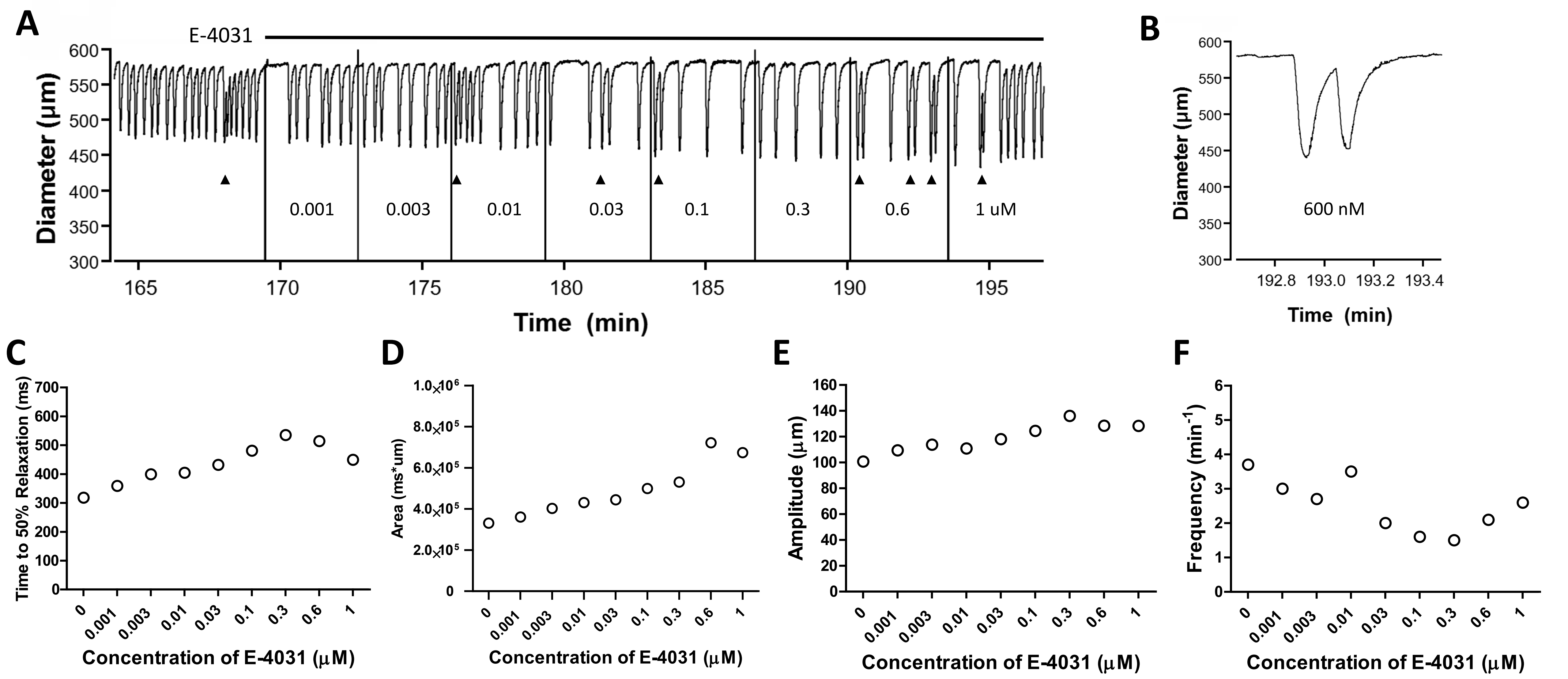

Supplemental Fig. 5. Effects of E-4031 on spontaneous contractions of a human pressurized mesenteric lymphatic vessel. **A**) Time course of changes in diameter and contraction pattern with increasing concentrations of E-4031. **B**) Example of double contraction in response to E-4031. Double contractions, which were occasionally observed in human vessels even in the absence of the ERG-1 inhibitor, are marked by arrowheads in **A**. **C-F**) Contraction parameters show same general trends as for rat mesenteric lymphatics (**Fig. 4**): increase in: time to 50% relaxation (**C**), area under the diameter-time curve (**D**) and amplitude (**E**); decrease in frequency (**F**).
