# Supplementary material for: ERG K+ channels mediate a major component of action potential repolarization in lymphatic muscle
Source: Sci Rep. 2023 Sep 9;13:14890. doi: 10.1038/s41598-023-41995-5 (PMC10492848; doi:10.1038/s41598-023-41995-5)
Supplement: Supplementary file 7 — Supplementary Figure 6. [file 41598_2023_41995_MOESM7_ESM.pdf]

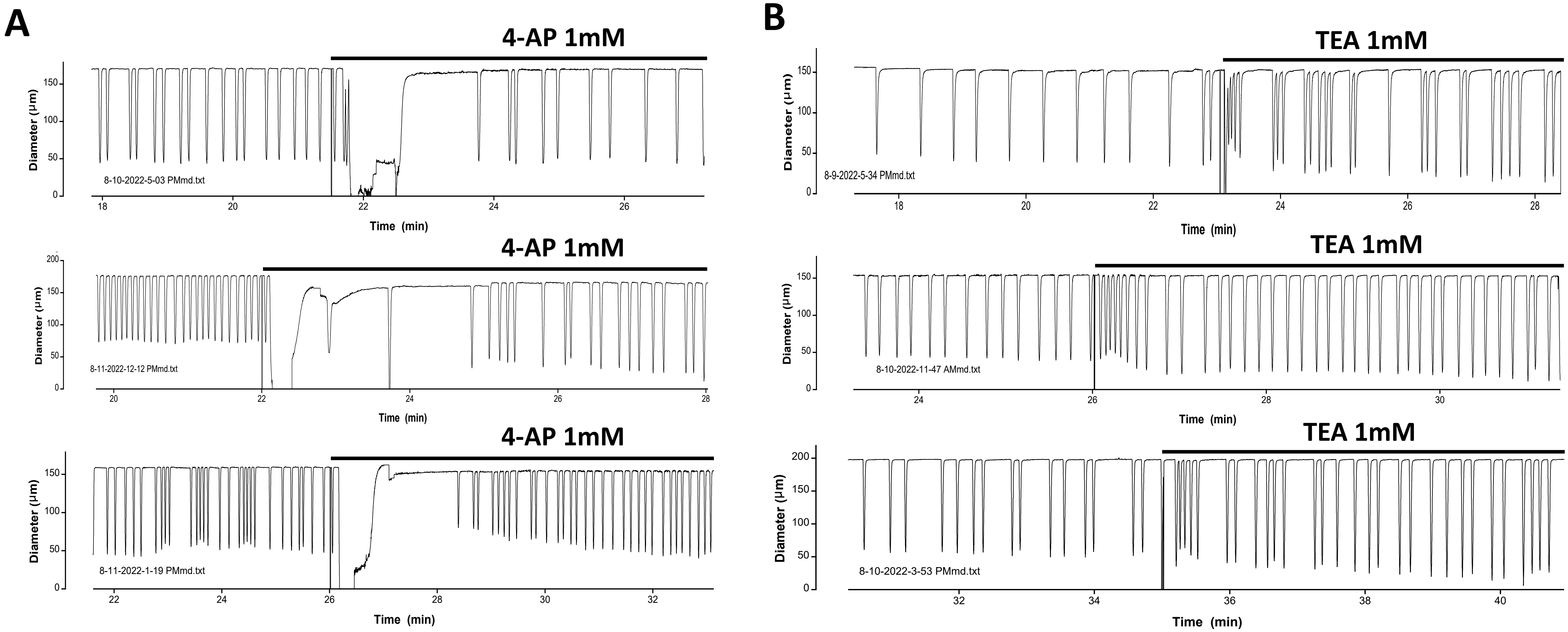

Supplemental Fig. 6. **A**) Three representative examples of the effects of 4-AP (1 mM) on the spontaneous contraction pattern of rat pressurized mesenteric lymphatic vessels. Immediately after 4-AP addition, contractions ceased for 10-20 sec and then resumed at (usually) a lower rate than control. **B**) Three representative examples of the effects of TEA (1 mM) on the spontaneous contraction pattern of rat pressurized mesenteric lymphatic vessels. Immediately after TEA addition, contraction frequency accelerated before stabilizing at a frequency higher than control.
