# Supplementary material for: ERG K+ channels mediate a major component of action potential repolarization in lymphatic muscle
Source: Sci Rep. 2023 Sep 9;13:14890. doi: 10.1038/s41598-023-41995-5 (PMC10492848; doi:10.1038/s41598-023-41995-5)
Supplement: Supplementary file 8 — Supplementary Information 8. [file 41598_2023_41995_MOESM8_ESM.docx]

**Supplementary Methods**

***Human tissue procedures***. Protocols on human lymphatics were conducted as approved by the Human Research Protection Office at Washington University (protocol #201111038 to GJR) and conformed to the principles of the Declaration of Helsinki. Discarded samples of mesenteric fat and sometimes gut wall were collected from de-identified consenting patients undergoing intestinal surgical resections for bowel inflammation, obstruction, cancer or polyps at Washington University Barnes-Jewish Hospital. Staff affiliated with the Washington University Digestive Diseases Research Core Center provided informed consent, tissue collection oversight, and de-identification services as per protocol. Tissue was placed immediately in cold physiological saline and shipped overnight from Saint Louis to Columbia, MO. Upon arrival, any remaining fragments of mucosal were removed and the sample was rinsed and stored at 4^o^C in Krebs-BSA. Lymphatic vessels processed in this way were viable for up to 72 hrs after surgery, as judged by their ability to develop spontaneous contractions when isolated and prepared for either pressure or wire myography. Human mesenteric lymphatics used for RT-PCR were snap frozen in liquid N_2_ after dissection and stored at -80^o^C until use.

***Human vessel isolation and force recording.*** The fragile valve sinuses of human mesenteric lymphatic collectors typically developed leaks upon cannulation and pressurization, preventing their routine use by pressure myography. For this reason, we used wire myography for most human vessel protocols ^47,48^. After dissection from the mesenteric fat and cleaning, two pieces of 40 μm stainless-steel wire were passed through the vessel lumen, in the direction of normal flow, and the vessel + wire was transferred to the chamber of a wire myograph (Model 310 A, Danish Myo Technology, Aarhus, DK) where the wires were clamped in the myograph jaws. Segments ≤1 mm in length were used to facilitate the maintenance of isopotentiality. The segment was stretched to approximately its optimal preload and the upper surface of the vessel was cleaned of fat and loose connective tissue. After heating to 37^o^C in Krebs-BSA buffer, viable segments developed spontaneous force transients after 90-120 min.

***Vm recording in human lymphatics.*** We attempted Vm recordings from over 20 preparations of human lymphatic muscle. Many samples of human mesenteric fat contained only precollectors without a smooth muscle layer or contained collectors that, for unknown reasons, never developed spontaneous contractions after isolation and pressurization. In vessels that did develop spontaneous contractions, the application of wortmannin to blunt those contractions and permit Vm recordings, even at lower wortmannin concentrations than used in rat lymphatic protocols, abolished spontaneous contractions and APs. Thus, we were unable to successfully maintain Vm recordings from the LMC layer in any pressurized human vessels during additions of ion channel modulators to the perfusate, despite being able to do so in past studies ^34^. For these reasons we turned to the wire-myograph technique, which permitted the study of vessels with small holes and, because the preparations were theoretically isometric, did not require wortmannin. Under these conditions 9 of 10 collectors developed spontaneous contractions. We were able to make Vm measurements in many of those vessels, but the electrodes nearly always dislodged during repetitive solution changes. We encountered other problems as well. The short segments used to facilitate isopotentiality were nearly isometric in the circumferential direction but exhibited variable amounts of axial shortening that tended to dislodge Vm electrodes. In addition, the basal frequency of wire-myograph-mounted human mesenteric lymphatics was unusually low, even at higher preload levels, and a successful impalement often resulted in cessation of all activity for a prolonged period of time—a single contraction over a 30 min period was not unusual. The reasons for this collective behavior are not known but may have been related to the necessity of shipping and cooling the human vessels until they were mounted in the myograph, which resulted in a 3-4x longer time than required by rat or mouse vessels (dissected at room temperature) to develop spontaneous activity after heating to 37^o^C.

**Supplementary Figure Legends**

**Supplemental Fig. 1**. **A**) Vm recording in an LMC along with diameter recording of a rat pressurized mesenteric lymphatic (after wortmannin treatment), showing that 1 μM E-4031 led to double spikes in half of the APs, corresponding in at least two cases with double contractions (arrowheads). **B**) Vm recording in an LMC of another rat pressurized mesenteric lymphatic in which 1 μM E-4031 led to three spikes.

**Supplemental Fig. 2**. Time and vehicle controls for E-4031 and BeKm-1. **A**) Recording showing time control with addition and mixing of vehicle (Krebs solution without ERG inhibitor) at ~2-min intervals. **B**) Insets show representative APs at expanded time scale for each time interval. Summary of changes in AP plateau duration (**C**) or normalized AP plateau duration (**D**) over time. Error bars are ± SD. There were no significant differences from control in either C or D, using a one-way ANOVA with Dunnett’s post-hoc tests. N= 4; n = 6.

**Supplemental Fig. 3**. Time and vehicle controls for ICA-105574 and RPR-260243. **A**) Recording showing time control with addition and mixing of vehicle (DMSO, at equivalent concentrations used for the ERG channel activators but without the activator) at ~2-min intervals. **B**) Examples of individual APs on an expanded time scale (a-g) at the corresponding time points indicated in panel A. Summary of changes in AP plateau duration **C**) or normalized AP plateau duration (**D**) over time. Error bars are ± SD. There were no significant differences from control in either **C** or **D**, using a one-way ANOVA with Dunnett’s post-hoc tests. N= 3; n = 6.

**Supplemental** **Fig. 4**. Vm recording from a human pressurized lymphatic vessel showing progressive widening of the AP plateau, with multiple spikes, in response to increasing concentrations of E-4031.

**Supplemental** **Fig. 5**. Effects of E-4031 on spontaneous contractions of a human pressurized mesenteric lymphatic vessel. **A**) Time course of changes in diameter and contraction pattern with increasing concentrations of E-4031. **B**) Example of double contraction in response to E-4031. Double contractions, which were occasionally observed in human vessels even in the absence of the ERG-1 inhibitor, are marked by arrowheads in **A**. **C-F**) Contraction parameters show same general trends as for rat mesenteric lymphatics (**Fig. 4**): increase in: time to 50% relaxation (**C**), area under the diameter-time curve (**D**) and amplitude (**E**); decrease in frequency (**F**).

**Supplemental Fig. 6**. **A**) Three representative examples of the effects of 4-AP (1 mM) on the spontaneous contraction pattern of rat pressurized mesenteric lymphatic vessels. Immediately after 4-AP addition, contractions ceased for 10-20 sec and then resumed at (usually) a lower rate than control. **B**) Three representative examples of the effects of TEA (1 mM) on the spontaneous contraction pattern of rat pressurized mesenteric lymphatic vessels. Immediately after TEA addition, contraction frequency accelerated before stabilizing at a frequency higher than control.
